# Utility values and electronic device use in low-vision people attending rehabilitation services: Data from a nation-wide registry in Italy

**DOI:** 10.1371/journal.pone.0308569

**Published:** 2024-08-09

**Authors:** Gianni Virgili, Eliana Costanzo, Ilaria Biagini, Mariacristina Parravano, Alessia Di Simone, Filippo Amore, Mauro Tettamanti, Simona Di Pietro, Giovanni L. Ciaffoni, Giovanni Sato, Giovanni Giacomelli, Federico Bartolomei

**Affiliations:** 1 Department Neurofarba, University of Florence, Florence, Italy; 2 IRCCS–Fondazione Bietti, Rome, Italy; 3 Unione Italiana dei Ciechi e degli Ipovedenti Onlus-Sezione Territoriale di Enna, Enna, Italy; 4 National Centre of Services and Research for the Prevention of Blindness and Rehabilitation of Visually Impaired, IAPB- Italia Onlus, Rome, Italy; 5 Fondazione Policlinico Universitario "Agostino Gemelli" IRCCS, Rome, Italy; 6 Istituto di Ricerche Farmacologiche Mario Negri IRCCS, Milan, Italy; 7 Azienda Ospedaliera Policlinico Tor Vergata, Roma, Italy; 8 Istituto dei Ciechi F. Cavazza, Bologna, Italy; 9 Centro Oculistico S. Paolo Hospital, S. Antonio Padova, Padova, Italy; University of Naples Federico II, ITALY

## Abstract

**Purpose:**

To estimate utility values associated with visual loss using EuroQol (EQ-5D) questionnaire, the impact of low-vision (LV) device use on utilities and the contribution of Instrumental Activities of Daily Living (IADL) score in patients attending vision rehabilitation (VR) services enrolled in the Italian Device & Aids Register (D.A.Re).

**Methods:**

This is a multicenter, prospective, cross-sectional study. D.A.Re. collects general and clinical information, vision-specific variables, use of electronic devices and quality of life questionnaires.

**Results:**

A total of 442 patients (75.0±16.6 years, 275 female) were included, 88 (19.9%) used specialised electronic LV devices, and 116 (26.2%) used smartphones and tablets. Users of smartphones and tablets were younger than non-users (67.5 vs. 77.6 years, p<0.001), but overall, their age ranged between 20 and 93. Stronger associations were found between vision-specific variables and IADL score compared to EQ-5D score. In multivariable age-adjusted models, the utility value of using smartphones and tablets on EQ-5D score was 0.12 (p<0.01), slightly larger than that of 1.0 logMAR difference (-0.09, p<0.01) or visual field damage within 10° of fixation (-0.10, p<0.01). Use of portable low-vision electronic devices and being employed or student (vs. retired) was also associated with better utility values (0.12 and 0.15, respectively, p<0.05).

**Conclusions:**

Visual loss is associated with loss of utilities in Italian patients attending VR services, whereas special-purpose electronic aids, and smartphone and tablet use are associated with better utility values. We found that IADL may be more sensitive to visual loss than EQ-5D and could be a valid health-related quality of life outcome in trials on VR.

## Introduction

Central to Value-Based Medicine is the concept that all patients deserve the interventions that provide the greatest patient value, in terms of improvement in quality of life (QoL) and/or length of life) [1]. Utilities could be useful and challenging tools for quantifying QoL in order to provide a standardization for a QoL preferences, even if controversies remain between societal and patients utilities [1, 2]. Estimating utility values in patients with visual impairment (VI) is crucial for evaluating the impact of loss of vision on their overall health-related quality of life (HRQoL). While no single HRQoL measurement tool is considered the gold standard, different instruments can produce utility scores that are remarkably consistent with each other [3].

A widely used tool to estimate HRQoL and derive utilities is the EuroQol (EQ-5 dimension, 5D) questionnaire, a generic instrument developed to measure health outcomes [4] across different populations and medical conditions, integrating several attributes [1, 2], exploring five dimensions: mobility, self-care, usual activities, pain/discomfort, and anxiety/depression [5].

Several studies have employed the EQ-5D to assess utility values in patients with VI, including those with age-related macular degeneration (AMD), diabetic retinopathy (DR), and glaucoma [3–6]. A recent meta-analysis on a total of 30,491 participants reported that patients with different eye diseases (such as AMD, DR, glaucoma and cataract) tended to have lower EQ-5D scores than healthy subjects [4]. Regarding visual loss, the EQ-5D can capture its impact on daily functioning, mobility, and self-care, but it may be less sensitive to the other two domains, anxiety/depression, and particularly pain [3]. As a result, in some studies, the EQ-5D lacked sensitivity for ocular diseases and did not show a linear correlation with vision [1, 2, 7]. Recently, two systematic reviews have established a link between visual loss and utility values estimated with the EQ-5D, finding a variable sensitivity of this questionnaire to different levels of visual loss [3, 4].

The EQ-5D utility values are used in economic evaluations by Regulatory Bodies, such as National Institute for Health and Care Excellence (NICE), including in cost-effectiveness analyses, to assess the relative value of interventions and healthcare technologies [2]. Poor sensitivity of EQ-5D to visual loss could be a disadvantage for health professionals promoting and investigating vision rehabilitation (VR) [5]. The utility metrics is important as the basis of Quality-Adjusted Life-Yars (QALYs), considering that vision disorders exert a big impact on QoL, but rarely impact lifespan, and despite the large heterogeneity across studies and small sample sizes, even small QoL impairments could result in great loss on the whole [4, 8]. Concerning EQ-5D, country-specific value sets have been developed for both versions (-3level, L and -5L) in a number of countries to reflect the health preferences of local general populations [4].

In Italy, in 2019 the National Institute for Device and Technology Assessment (Istituto Nazionale Valutazione Ausili e Tecnologie, INVAT), developed the Device & Aids Register (D.A.Re) to collect data from low-vision (LV) people in different VR services, integrating information from technology and aids, vision performance and the QoL questionnaires [9]. VR is offered to patients to teach how to cope with irreversible visual loss and its consequences on daily life, including social and psychological issues. We have previously shown that the use of LV aids (LVAs), particularly electronic devices, as well as of smartphones and tablets, is associated with better HRQoL measured with the Instrumental Activities of Daily Living (IADL) questionnaire [9, 10]. Having used the IADL score to successfully quantify activity-based QoL in patients attending VR services [9–12], we considered linking this evidence to utility estimates, which are important in economic evaluations as a useful further step of development of D.A.Re.

The aims of the present study were: 1) to estimate utility values associated with visual loss using the EQ-5D questionnaire in a consecutive sample of patients attending VR services in Italy included in D.A.Re; 2) to investigate the impact of electronic LV device use on utilities; 3) to assess the contribution of IADL to utility estimates in these patients.

## Materials and methods

This is a multicenter, prospective study, exploiting the network of Italian VR services affiliated with the Italian Union for the Blind and Visually Impaired (Unione Italiana Ciechi e Ipovedenti, UICI), under the coordination of the University of Florence. The study protocol was approved by the Ethics Committee of the Area Vasta Centro-Careggi, Florence at 19^th^ May 2020 (#15992_OSS CEAVC Comitato Etico di Area Vasta Centro della Toscana). The recruitment in D.A.Re started the 3^th^ June 2020 and is ongoing. For the present study we analyzed data of patients who completed the EQ-5D questionnaire between January and June 2023.

Written informed consent was obtained for all patients. Inclusion criteria were: i) patients aged 18 years or older, affected by any visual pathology, attending specialized centers for visual education and rehabilitation for VI and/or orientation and mobility with personal autonomy training; ii) ability to understand and sign the informed consent to participate in the study; iii) sufficient mastery of the Italian language and cognitive capability to engage in an interview or questionnaire. Exclusion criteria were: i) children under 18 years; ii) cognitive impairment that, at the experimenter’s discretion, could have limited the understanding of informed consent or the questionnaire.

As previously published (9, 10), D.A.Re is a nation-wide registry collecting data from 20 VR services in 10 Italian regions, providing a picture of patients attending VR services, including their general characteristics, occupational status, blind registration status, general knowledge and use of computers and software, integrated with clinical information about ocular and systemic diseases, in particular hearing impairment. Vision-specific variables are visual acuity (VA), maximum reading speed (MRS)(measured binocularly at 20 cm with appropriate correction by the Minnesota low vision reading [MNREAD] chart), critical print size (CPS) and type of VI (including visual field restriction) and, in addition, several details about the use of LVAs, knowledge of Braille, numerous details on type, brand and scope of use of LV devices in use (optical, electronic or others), in particular for electronic devices, technological details as screen size, touch-screen and Optical Character Recognition (OCR) functions, text-to-speech function, or other specific functions. In addition, the EQ-5D and IADL questionnaire scores were calculated for each included patient, to define their levels of autonomy. All data from D.A.Re. are anonymously stored in a server owned by INVAT; their use has been approved by the local Ethics committee (#15992_OSS CEAVC Comitato Etico di Area Vasta Centro della Toscana). For the present analysis data were extracted on 25^th^ July 2023.

### Health-related quality of life instruments

The IADL questionnaire is a validated method to measure functional health and QoL in different clinical situations [11, 12]. Previous evidence indicates that the evaluation of these dimensions helps to identify problems that require treatment or care [13]. The IADL score has been routinely collected in D.A.Re since inception, and consists of eight questions regarding daily activities defining different levels of self-efficacy and autonomy for each question [10]. A low IADL score indicates good autonomy [9]. This validated questionnaire consists of eight questions regarding daily activities such as telephone use, shopping, cooking, housekeeping, laundry, use of public transport, responsibility for drug taking, financial management [10]. Different aspects of daily life are explored by the IADL questionnaire, helping to identify problems requiring treatment or care [13].

In this study, we prospectively adopted the EQ-5D questionnaire as a means for computing utility values attached to various levels of visual loss, exploring the potential association with individual characteristics and the use of LV aids, particularly electronic devices; among these, tablets and smartphones were considered separately from special-purpose electronic devices (i.e. hand-held and stand Closed Circuit TV), as they are general purpose devices. Both, EQ-5D and IADL questionnaires were administered during the visit by the VR service team.

### Statistical analysis

For descriptive purposes, we presented boxplots by users vs non-users of special-purpose electronic devices, or smartphones and tablets. Comparisons between these groups were conducted using Wilcoxon rank sum test.

Regarding our objective #1 (to estimate utility values associated with visual loss with the EQ-5D questionnaire in patients attending VR services in Italy) and #2 (to estimate the impact of LV devices use on utilities in these patients), we used univariable and multivariable linear mixed models with EQ-5D as a dependent variable. Mixed models accounted for within-subject correlation in individuals using more than one low-vision device, which were recorded as multiple rows per subject in the dataset. In regression models, we used the R-square parameter to estimate the proportion of variance of EQ-5D or IADL scores that is explained by visual function variables accounting for confounders.

Regarding objective #3 (to investigate the contribution of IADL to utility estimates), we investigated the shape of the association between EQ-5D and IADL score using locally weighted regression, and then conducted mediation analysis using Structural Equation Modelling (SEM) (StataCorp. 2023. Stata 18 Structural Equation Modelling Reference Manual. College Station, TX: Stata Press) [14], in which covariates of interest, as identified in previous studies based on D.A.Re, had direct effects on EQ-5D score, as well as indirect effects mediated by IADL score. The goal of these exploratory analyses was to investigate the sensitivity of these HRQoL tools to visual functioning.

A post-hoc power calculation of the power to detect a clinically meaningful difference in utility score was conducted on users (n. 116) vs. non-users (n. 326) of smartphones and tablets with the following values: means 0.74 and 0.53, standard deviations: 0.22 and 0.38, respectively. We found that our study had 93% power to detect a difference in utility values of 0.1 or larger, and a 78% power to detect a difference of 0.08, with alpha = 0.05. We considered these minimally important difference (MID) values based on Henry et al., [15] who conducted world-wide estimates of MID for EQ-5D utilities, among which we used values observed in Germany and Ireland as Europe-based estimates.

All analyses were conducted using Stata 18.0 software (StataCorp, College Station, TX).

## Results

We included 442 patients with mean age 75.0 years (SD: 16.6; 48.4% 80+ and 8.5% 90+), of whom 275 were female (62%). The ocular conditions associated with VI in our series were: AMD 61.4%, DR 2.7%, glaucoma, 7.8%, cataract 0.5%, inherited retinal dystrophies 10.3%, high myopia 11%, optic nerve atrophy 2%, hemianopsia 1.8%, other 2.5%. The median value (IQR) of EQ-5D score was 0.69 (0.41–0.85) and that of IADL score was 3.92 (2.65–5.13).

Of 442 patients, 354 (80.1%) did not use any electronic device, 42 (9.5%) used portable special-purpose electronic devices only, 40 (9.0%) used stand devices only, and 6 (1.4%) used both. Women were more likely to use electronic devices than men (22.6% vs 15.6%), a difference approaching but not crossing nominal statistical significance (p = 0.075). There was no difference in employment status and visual field restriction for users vs non-users of special-purpose electronic devices.

**Fig 1:**
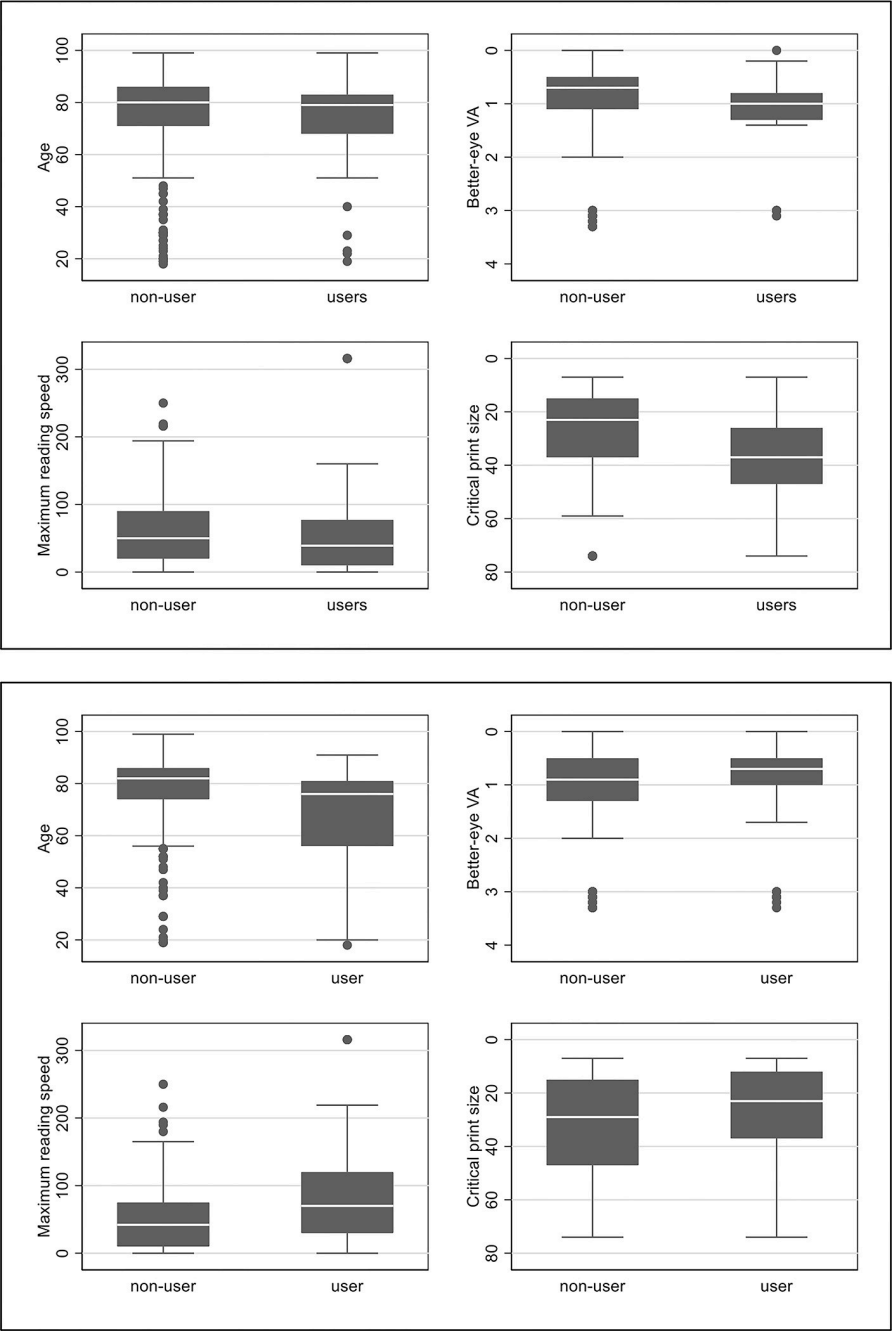
1a (top panel) and 1b (bottom panel). Boxplots of age (top left in each panel), better-eye visual acuity (top right in each panel), maximum reading speed (bottom left in each panel) and critical print size (bottom right in each panel) for users and non-users of electronic devices (Fig 1A) or users and non-users of smartphones and tablets (Fig 1B).

As seen in the Fig 1A, the age of users of special-purpose electronic devices was only slightly inferior to that of non-users, with no statistically significant difference (median [IQR]: 73.9 68–83] years vs. 80 [IQR = 71–86] years (p = 0.137). Of note, better-eye VA was slightly worse for users of special-purpose electronic devices (1.0 [0.8–1.3] logMAR vs 0. 7 [IQR: 0.5–1.1] logMAR, p<0.001), as was the critical print size (CPS, mean: 37.0 [26–47] vs 23 [15–37] points, p<0.001), but Maximum Reading Speed (MRS) tended to overlap (39 [10–77] wpm vs 50 [IQR: 20–90] wpm, p = 0.097; Fig 1A).

One-hundred and sixteen (26.2%) patients used smartphones and tablets, of whom 38 (32.8%) also used a special-purpose electronic device. More users of smartphones and tablets were employed/students (23.5% vs 5.5%, p<0.001), while non-users had more often visual field damage (21.8% vs 6.9%, p = 0.001). This may have been due to the fact that users of smartphones and tablets were younger than non-users (median [IQR]: 76 [56–81] years vs. 82 [74–86] years, p<0.001), but overall, the users’ age extended over a wide range between 20 and 93 years (Fig 1B). Better-eye VA largely overlap for users and non-users (0.7 [0.5–1.0] logMAR vs 0.9 [0.5–1.3] logMAR, p = 0.125), but MRS was much better for users vs non-users (70 [30–120] wpm vs 42 [10–75] wpm, p<0.001), as was CPS (23 [12–37] points vs 29 [15–47] points, p = 0.004). S1 Fig shows the age distribution of electronic aids users in detail as a histogram.

### Study aim #1 and #2: Utility values associated with VI and electronic device use

**Fig 2:**
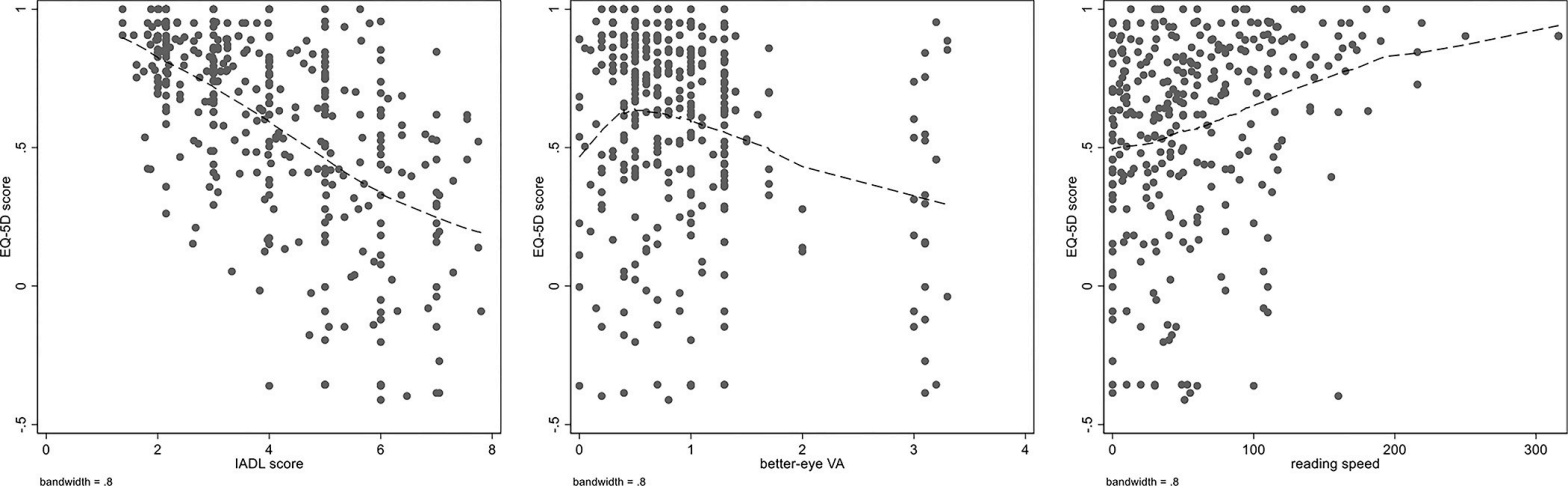
Relationship between EQ-5D score and IADL score, better-eye visual acuity (VA) and reading speed. Dispersion graph with lowest smoothing for the relationship between EQ-5D score and IADL score, better-eye VA (in LogMAR) and reading speed (in words/minute).

We explored which variables of interest (age, VA, visual field restriction, device screen width, employment status, optical aids, hearing impairment, smartphone/tablet use), as identified in our previous study (9, 10), were associated with EQ-5D score. As expected (Table 1), multivariable associations with EQ-5D score were diluted compared to univariable associations, and remained significant for better-eye VA, visual field restriction, use of small-screen electronic devices compared to no device, and being employed or student compared to being retired. All these covariates were associated with at least 0.1 utility score differences for dichotomous or nominal covariates, and, similarly, with a difference of 1.0 logMAR VA (Table 1). Fig 2 also shows that the relationship between IADL and EQ-5D score was linear.

**Table 1:** Univariable and multivariable regression coefficients between covariates and EQ-5D score (columns 1 to 2); SEM model investigation mediation of covariate effects on EQ-5D score by IADL score (columns 3 to 5).

	Regression model with EQ-5D as response variable		SEM model investigation mediation of covariate effects on EQ-5D score by IADL score		
variable	1. EQ-5D	2. EQ-5D	3. IADL	4. EQ-5D	5. EQ-5D
	Univariable	Multivariable	SEM	SEM	SEM
			multivariable	indirect	direct
IADL score					-0.116**
Age (10 y)	-0.054**	-0.003	0.002	0.000	-0.001
Visual acuity	-0.122**	-0.089**	0.787**	-0.091**	0.000
(1.0 logMAR)					
Visual field restriction	-0.220**	-0.098*	-0.629**	-0.072**	-0.0252
Device screen width (inches, reference: no device)	1	1	1	1	1
5-	0.135**	0.123*	-0.818**	0.089*	0.034
6–18	0.186*	0.085	-1.102*	0.120	-0.036
19+	-0.036	0.030	-0.192	0.029	0.001
Employment retired	1	1	1	1	1
employed/ student	0.263**	0.150*	-1.038**	0.102*	0.048
other	0.073	0.140	-0.276	0.030	0.017
Optical aids	-0.004	0.035	-0.009	0.001	-0.017
Hearing impairment	-0.134**	-0.051	0.458*	-0.053	0.002
Smartphone/ tablet	0.210**	0.115**	-1.04**	0.120**	-0.005

*: p<0.05

**: p<0.005 (Bonferroni adjusted for multiple comparisons)

### Study aim #3: Contribution of IADL score to utility value assessment

We found stronger associations between our variables of interest and IADL score compared to EQ-5D score. In fact, the proportion of variance (R-square) explained by better-eye VA alone was 13% for IADL and 6% for EQ-5D; the proportion of variance explained by all covariates in Table 1 was 38% and 18%, respectively, thus nearly twice as much for IADL compared to EQ-5D scores. Moreover, using SEM for mediation analysis, the effect on utilities score of VA, visual field restriction, special-purpose electronic devices with small screen, being employed/student, and using smartphone or tablets was largely indirect and mediated by IADL score (Table 1). As an example, the estimated utility value of smartphone and tablet use was entirely mediated by IADL score, indirect effect = 0.120 utility vs. direct effect = -0.005.

The EQ-5D subscales contributed similarly to the overall score, with correlation coefficients between 0.65 for anxiety and 0.74 for mobility (p<0.001).

### Can we estimate utilities from IADL score?

Our findings suggest that IADL questionnaire is sensitive to visual factors as an HRQoL tool and is a valid mediator of the effect of visual loss and LV aids use in people with VI. In our SEM model, one-unit IADL increase corresponded linearly to -0.116 worse EQ-5D utility score. As presented above, using locally weighted regression the relationship of reading speed with utility was linear, whereas that with VA deviated at near-normal VA values, where patients were disabled due to visual field defects (Fig 2). These findings suggest that, in patients with VI attending VR services, utility values, a measure of HRQoL, used in economic models, could be derived from IADL score, with the advantage of a better incorporation of visual and demographic covariates.

## Discussion

In this study, we have estimated utility values, using EQ-5D, associated with visual loss in Italian patients attending VR services. In addition, we have investigated the impact of the use of electronic LV aids on utilities and the contribution of IADL to utility estimates.

Regarding utility values, we found that a difference of one ETDRS line of vision (0.1 logMAR) corresponds to -0.012 utility loss using EQ-5D. This is close to estimates made by Brazier et al., [16] in patients with diabetic macular oedema, among the causes of visual loss in our series, treated with intravitreal injections of aflibercept. Kai et al., [4] found that AMD, DR and glaucoma accounted for -0.03 to -0.06 utility loss; however, they did not consider disease severity or VA.

In a systematic review by Purola et al., [3] vision loss was mostly associated with dimensions related to physical health and capability, whereas the association with mental dimensions remained somewhat uncertain. This may support our finding of a stronger association between the covariates of interest and IADL score, which is based on perceived difficulty in life tasks, with respect to EQ-5D score. This topic remains debated and other authors found that EQ-5D-3L was unresponsive to LV rehabilitation, assessing a limited spectrum of domains, being instead sensitive to patients’ comorbidities [5, 17, 18].

It has been suggested that cost-effectiveness studies are necessary to understand the effectiveness of current VR practices [19]. Without evidence of cost-effectiveness of interventions intended to tackle the burden of vision loss, VR may be assigned limited resources, or resources might be allocated in an inadequate manner. The extraction of utility values for use in economic modelling follows different methods in different countries; for example in the United States health utilities are usually derived from patients’ evaluation or using VA as a surrogate outcome, whereas in the United Kingdom utilities are preferably derived from the public [8]. This means that health care decision makers could take into account different preferences to use/allocate resources [8]. Despite this, regarding EQ-5D estimate of utility values, a remarkable similarity was found across different studies worldwide expressing the global burden of vision loss [3].

Confirming the impact of vision loss on daily living tasks, our study suggests that IADL is more sensitive than EQ-5D to demographic and psychophysical variables and may give a better representation of the profile of people attending VR services, as compared to estimating utilities from VA score. In fact, we suggest that EQ-5D derived utility values can be estimated from IADL score in the broad populations of patients attending VR services, since their relationship is linear and IADL score is an effective mediator of visual functioning and device use. On the other hand, in our sample of diverse LV patients, better-eye VA was not linearly associated with EQ-5D score at near normal visual acuity levels, where visual field restriction plays a role, and this was likely due to genetic retinal disease and glaucoma. Overall, we found that one IADL score unit corresponds to 0.12 utility difference.

We found the use of portable special-purpose electronic devices, as well as of smartphones and tablets, was associated with EQ-5D and IADL scores that were larger than the effect of 1 logMAR VA (10 lines of vision), hearing impairment, and being employed or student in multivariable models. Optical aids use was not associated with different scores compared to no use. Smartphone devices are used by individuals with various levels of visual loss, age and diagnoses for different tasks, including face recognition, television or movie watching, near reading, computer screen use, distance reading and letter writing [20]. Abraham et al. [21] found that a significant number of people with severe vision impairment or blindness used smartphones, though most were unaware of its full functionality and assistive capabilities, as shown during VR. Moreover, a number of free apps are available, including apps to improve remote orientation and mobility instruction [22, 23].

Of note, in Italy the use of IADL score integrates the instance for subsidiarity/civil accompanying allowance according to the Law 122/2010 art 29-ter.

Our study has limitations, the first of which is its cross-sectional design, meaning that association, rather than causality, is detected. Multivariate adjustment, including demographic and social determinants, may strengthen our analyses, which help to identify candidate individual targets or interventions to be considered when planning longitudinal studies. In the review by Purola et al., [3] six studies showed VI or blindness to have an equal or stronger impact on HRQoL than major medical conditions. The lack of adjustment for major comorbidities is a limitation of our registry, since we only used data on hearing impairment, which has a large impact on QoL, whereas data on systemic conditions were not standardised. We plan to introduce the Charlson Comorbidity Index and an instrument assessing cognitive impairment to fully account for this in D.A.Re, and also to use the registry to conduct longitudinal studies.

Finally, we confirm our previous findings of the key role of special-purpose portable electronic aids and, especially, smartphone and tablets in QoL, as perceived by people with vision impairment, finding an effect not only on IADL score, but also on EQ-5D score. We may have detected ‘happy user’ profiles rather than causality, given the cross-sectional nature of our registry, which we tried to compensate with adjustment by functional, social and demographic determinants. Nevertheless, our findings support the conduction of further research on the implementation of the use of electronic aids, smartphones and tablets in LV people. Furthermore, we also plan to obtain longitudinal data on the provision of these devices to assess the potential for conducting trials which compare models of VR care.

In conclusion, we measured utility values associated with visual loss in Italian patients attending VR services, as well as those associated with electronic technology use for VR, accounting for socio-demographic factors. We found that IADL is a major determinant of utility, and may be more sensitive to visual loss than EQ-5D. Thus, IADL could be a valid HRQoL outcome in trials on a VR.

## Supporting information

S1 Fig Histogram of the age distribution of electronic aids users. (PDF)
