# Supplementary figures and images for: Utility values and electronic device use in low-vision people attending rehabilitation services: Data from a nation-wide registry in Italy
Source: PLoS One. 2024 Aug 9;19(8):e0308569. doi: 10.1371/journal.pone.0308569 (PMC11315328; doi:10.1371/journal.pone.0308569)

special-purpose electronic devices

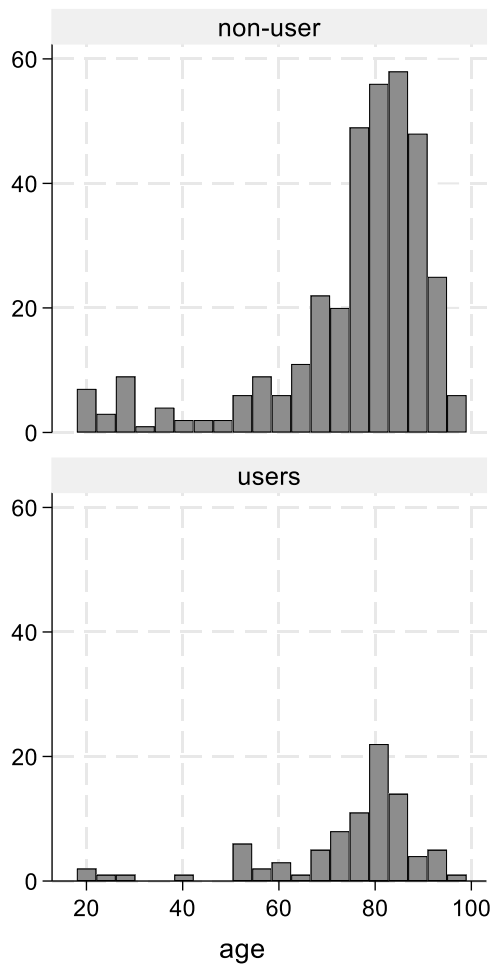

smartphones and tablets

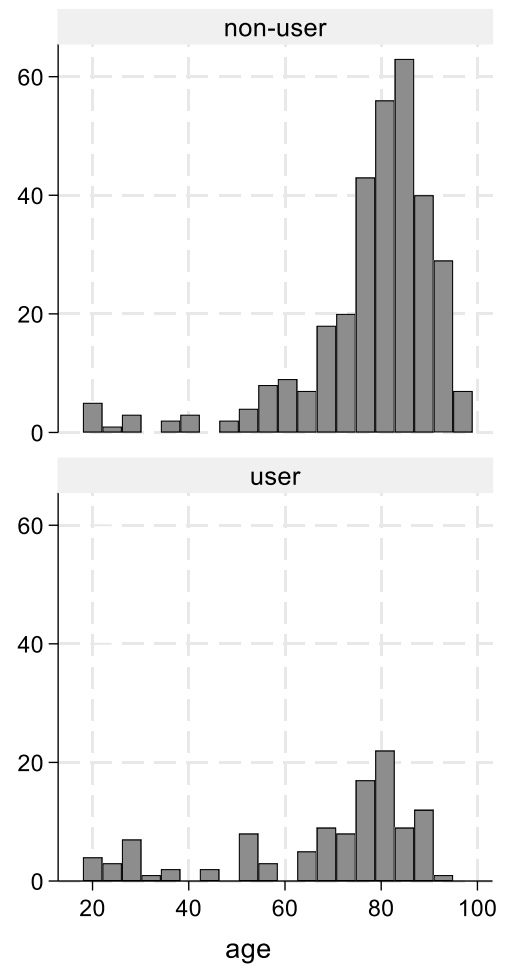

Supplement: S1 Fig — (PDF) [file pone.0308569.s001.pdf]
